# Different perspectives on a common goal? The Q-method as a formative assessment to elucidate varying expectations towards transdisciplinary research collaborations

**DOI:** 10.1007/s11625-022-01192-1

**Published:** 2022-08-06

**Authors:** Verena Radinger-Peer, Elisabeth Schauppenlehner-Kloyber, Marianne Penker, Katharina Gugerell

**Affiliations:** 1 Department of Economics and Social Sciences, Institute for Sustainable Economic Development, University of Natural Resources and Life Sciences, Vienna, Feistmantelstraße 4, 1180 Vienna, Austria; 2 Department of Landscape, Spatial and Infrastructure Sciences, Institute of Landscape Planning, University of Natural Resources and Life Sciences, Vienna, Peter-Jordan-Straße 65, 1180 Vienna, Austria

**Keywords:** Transdisciplinarity, Heterogeneity, Transdisciplinary research collaboration, Viewpoints, Q-method

## Abstract

Transdisciplinary research (TDR) collaborations are considered effective when they yield relevant results for science and practice. In this context, the different expectations, experiences, skills, and disciplines of the team members involved determine TDR collaboration. Using the example of 13 team members involved in the 3-year TDR project ‘Römerland Carnuntum 2040’ (Austria), we aim to identify and compare diverse expectations regarding TDR collaboration. In doing so, we question the often emphasised dichotomy between science and practice as the main challenge of TDR collaboration and aim towards making individual expectations regarding TDR collaboration visible and tangible. The contribution of the present paper is twofold: on the one hand, we provide statements for a formative assessment to externalise implicit expectations, assumptions, and epistemologies of TDR project team members regarding TDR collaboration and results. On the other hand, we present the Q-methodology as a viable approach to uncover diverging viewpoints as visible, tangible, and enunciable differences that need to be acknowledged in early stages of TDR projects when allocating resources and planning further project steps. Our investigations result in two viewpoints: one emphasises learning, collective reflection, and knowledge exchange as the main TDR expectation. The second focuses on ‘changing practices’, assuming that the project supports the introduction of new practices for (sustainable) regional development. These diverging expectations reveal subconscious tensions, which have to be addressed when allocating resources and defining project success within the TDR project.

## Introduction

Transdisciplinary research (TDR) promises to be particularly effective in responding to complex societal problems (Gibbons and Nowotny 2001) and in pursuing transitions towards a more sustainable society (Geels and Schot 2007; Hölscher and Frantzeskaki 2020; Köhler et al. 2019; Meadows 1999; Meadows and Wright 2008). In this sense, TDR collaborations are considered effective when they yield relevant outcomes for science and practice and involve both rigorous contributions to scientific progress and the co-creation of applicable and ‘workable’ solutions for practice (Binder et al. 2015; Gibbons and Nowotny 2001; Gibbons et al. 1994; Lang et al. 2012; van Drooge and Spaapen 2017). Hence, collaboration of academic researchers with diverse disciplinary backgrounds and non-traditional partners facilitates an inter-sector, problem-oriented, and demand-driven research approach, and is a fundamental requirement for TDR (Brown et al. 2010; Klein 2008; Paschke et al. 2019; Pohl 2008).

The TDR team members involved bring an essential heterogeneity of skills and expertise to the problem-solving process (Gibbons and Nowotny 2001). At the same time, TDR team members belong to different thought collectives (Fleck 1935), are socialised in varying (professional) cultures and languages with distinct epistemological paradigms (Hakkarainen et al. 2020; Scholz and Steiner 2015). Furthermore, they pursue different intentions and objectives (Boon et al. 2014; Hegger et al. 2012), and/or operate within highly divergent timeframes and institutional logics (Alonso-Yanez et al. 2019; Bergmann et al. 2021; Scholz and Steiner 2015; Thompson et al. 2017). The diverging expectations, experiences, skills, disciplines, and practices influence the collaboration in the team during the joint project. For these variables, previous research emphasises a dichotomy between scientists and practitioners in the TDR team (Lang et al. 2012; Scholz and Steiner 2015). However, reflections on different roles (by researchers, Wittmayer and Schäpke 2014) in sustainability transitions suggest a more diverse picture with different ‘shades of grey’ rather than a simple ‘black and white’ dichotomy.

While team diversity is expected to foster innovation processes (Cummings et al. 2013; Guimera 2005; Henneke and Lüthje 2007; Lungeanu et al. 2014), previous research stresses that diversity might render conflicts (Guimera 2005), and challenges the formation of a group identity. Group formation includes the development of a sense of belonging (‘we’ rather than ‘me, myself’) (Bergmann et al. 2021; Fam et al. 2020), a common language, a shared understanding of problems and research, and an appreciation of diverging epistemologies, norms, different forms of knowledge (Alonso-Yanez et al. 2019; Bergmann et al. 2021), and related integration processes. To facilitate these integration processes, the actors involved have to externalise their expectations, assumptions, and epistemologies (Hakkarainen et al. 2020) in a way that allows others to understand (organisational, knowledge-related, expected) boundaries of collaboration as well as (organisational, financial, knowledge-related) dependencies (Carlile 2004; Majchrzak et al. 2012). Only when expectations, boundaries, and dependencies emerge from the ‘subliminal’ to the surface, they become tangible, discussable, negotiable, and surmountable (Jahn et al. 2012; Majchrzak et al. 2012). Previous TDR has put notable effort in the development of formal, ex post summative assessment methods (Carew and Wickson 2010; Luederitz et al. 2017; Scholz and Steiner 2015; Wolf et al. 2013; Zscheischler et al. 2014). However, few of these methods quantify and statistically analyse expectations towards TDR collaboration and how its outcomes vary across TDR team members as formative assessments (exceptions are, e.g. Eigenbrode et al. 2007; O’Rourke and Crowley 2013).

This paper aims to fill this research gap and extend previous research (Alvarez et al. 2010; Mitchell et al. 2015, 2018; Thompson et al. 2017; van Drooge and Spaapen 2017) by investigating the following research questions: (1) how do expectations towards TDR collaboration and its outcomes vary across TDR team members?, (2) how can the Q-methodology make varying expectations of TDR collaboration visible and tangible? The paper focuses on the process and results of a Q-methodological approach implemented in the initial phase of the TDR project “Empowerment, self-organisation and regional transformation—the model of the Club of Rome Region Carnuntum” (Römerland Carnuntum 2040) with 13 TDR team members. Section 2 presents the conceptual background of the paper and the state of the art on formative assessments within TDR projects. Section 3 presents our case study and the overall project framework. In Sect. 4, we describe the methodological approach of the Q-method in detail, while Sect. 5 presents the achieved results. Section 6 discusses the findings, both in terms of their relevance to the management of TDR and the application of the Q-method.

## State of the art and conceptual background: formative assessment of varying expectations towards TDR collaboration among team members

Several studies emphasise a dichotomy between science and practice, and the mutual learning between these two realms as core characteristics of transdisciplinarity (Fam et al. 2020; Lang et al. 2012; Norris et al. 2016; Scholz and Steiner 2015). Thereby, TD acknowledges that science and practice refer to different epistemologies (i.e. ways of knowing) and reference systems (e.g. modes of validation, what is considered as good, adequate, or false) (Eigenbrode et al. 2007; Scholz and Steiner 2015). While Jahn et al. (2012) and Lang et al. (2012) juxtapose societal problems, societal discourse and results for societal praxis on the one side and scientific problems, scientific discourse and results for the scientific praxis on the other side in their framework of an idealised TDR process, Pohl et al. (2017) frame the dichotomy between science and practice as the “realm of science, rigour and understanding” and the “realm of practice, relevance and design”. Reviewing the discourse shows that scholars emphasise that the TDR process connects scientific knowledge production and societal problem handling via the formation of a common research object, the co-production of new knowledge and TD knowledge integration (Jahn et al 2012; Lang et al. 2012). It also emphasises that the two rationalities have to be met and balanced in the TDR process: the thought style of science searching for truth and the thought style of practice interested in workability (Pohl et al. 2017, based on Bergmann et al. 2005; Pohl and Hirsch Hadorn 2007; Jahn et al. 2012).

In our view, TDR project members of science and practice both hold relevant knowledge, interests in and power to influence the TDR process. However, based on previous empirical experience, we assume that the boundaries between science and practice are more blurred than outlined in TDR literature, and that further efforts are needed to elucidate and capture the individual expectations, skills, and perceptions of TDR team members that condition successful knowledge integration.

Team building plays an important role in initial project phases (Lang et al. 2012) and is critical for a successful team formation (Guimera 2005; Norris et al. 2016). Several studies (Angelstam et al. 2013; Lang et al. 2012; Norris et al. 2016; Scholz and Steiner 2015) emphasise the importance of this early phase to “(…) identify perspectives necessary to the success of the effort and then individuals who occupy those perspectives” (Norris et al. 2016, p. 116). At the same time, this process is challenged by various issues such as intra-group conflicts or feelings towards project managers or group members. Despite the many challenges of TD projects, in this paper we focus on the early stages of TDR collaborations and aim to elucidate those challenges that refer to the diverse expectations, assumptions and epistemologies (e.g. motivation, goals, project success and validation) of the involved team members.

Most recently, Fam et al. (2020) synthesised those debates into three core TDR tensions emerging between individuals and at team level: (1) I versus we: mismatch between individual and team (subliminal) expectations and assumed outcomes, a poor understanding of diverse expertise, and a lack of ‘we’ in the project; (2) disciplinary versus TDR: cognitive and epistemological differences, and a weak shared understanding of different research cultures and backgrounds involved beyond one's own (see Barth et al. 2020; Scholz and Steiner 2015); (3) research versus learning: tension to simultaneously learn and research, the need to negotiate rules and norms that guide the joint research process as well as struggles to conduct research in formless, comparatively ‘messy’ environments. Moreover, early TDR project phases are vulnerable in terms of communication, since this phase plays a pivotal role in team formation, establishing reciprocity (Bergmann et al. 2021; Cummings et al. 2013; Scholz and Steiner 2015; Wittmayer and Schäpke 2014; Zscheischler et al. 2014), and creating convergent views and relations (Angelstam et al. 2013; Putnam and Fairhurst 2001). Beech et al. (2010) emphasise meaningful, dialogic relationships as a basic requirement for TDR, but consider dialogues between practitioners and academics to be highly problematic, as they often consist of self-defense, with individuals unintentionally acting in ways that are seen in academic debate as a path to failure. Against this backdrop, Wiek (2007, p. 57) refers to the role of an ‘epistemediator’, acting as an intermediary between different actors, worldviews and perspectives, translating them, and facilitating “the (epistemic) process of joint knowledge generation”.

Formative ‘ex ante’ or ‘in situ’ assessments play an important role in the preparation and/or initial “team building” phases of TDR projects (Alvarez et al. 2010; Mitchell et al. 2018; van Drooge and Spaapen 2017). However, scientific knowledge about formative assessments “for learning”, which are important to unravel subliminal perceptions, tensions, expectations and epistemologies as well as develop adaptive measures for improving individual and/or team collaboration and performance (Majchrzak et al. 2012), is still fragmented and modest (Angelstam et al. 2013; Binder et al. 2015).

The TDR discourse presents different tools and approaches for formative TDR assessment. The ‘toolbox dialogue approach’ by Eigenbrode et al. (2007) as well as O’Rourke and Crowley (2013) represent early attempts to improve cross-disciplinary science by effecting epistemic changes that lead to better group communication. Both share the goal of making different epistemologies (see also Pohl and Wuelser 2019) of team members explicit (Eigenbrode et al. 2007) and to improve the effectiveness and efficiency of cross-disciplinary communication via interventions. A further contribution to uncover expectations and assumptions of the different team members and their thought styles is the ‘td-net toolbox’ for knowledge production (Pohl and Wuelser 2019).

Within our project and for the formulation of the Q-statements, the publications by Alvarez et al. (2010), Guimarães et al. (2019), Mitchell et al. (2018), Thompson et al. (2017), and van Drooge and Spaapen (2017) were particularly helpful because the formative assessments studied share a ‘begin at the end’ approach. That is, they focus on the expected and aspired outcomes of the TDR process right at the beginning of the TDR collaboration to derive implications for the process ahead. This is particularly true for the Participatory Impact Pathways Analysis (PIPA) approach (Alvarez et al. 2010; van Drooge and Spaapen 2017) and the Outcome Spaces Framework (Mitchell et al. 2015, 2018). Van Drooge and Spaapen (2017) propose PIPA as a means for evaluating different ideas, expectations, and assumptions of the actors involved at the beginning of a TDR process. PIPA should help find “common ground” among the actors involved, as it focuses on developing a shared understanding and a common sense of responsibility for the project and its intended impacts (van Drooge and Spaapen 2017). Mitchell et al. (2015) define three key realms of TDR outcome spaces: (1) the improvement within the situation or field of inquiry; (2) the generation of relevant knowledge assets and flows, including scholarly knowledge and other forms of societal knowledge, and making them accessible and meaningful to researchers, participants, and beneficiaries; and (3) mutual and transformational learning by scientists and research participants to increase the likelihood of persistent change, as this includes interpersonal and intra-team learning. We were further inspired by Thompson et al. (2017) and Guimarães et al. (2019): both emphasise the role of transdisciplinary researchers’ skills, motivations, attitudes, and behaviours that affect their expectations towards TDR as well as potential constraints in the team formation process. Thompson et al. (2017) expand the argument, referring to the need for a better understanding of project partners’ perspectives and expectations early in the TDR process. Using a TDR project to improve disaster resilience in New Zealand, they highlighted aspects of challenges, dialogue, integration, and benefits. They point out that a sequence of subliminal conflicts related to new formats of knowledge creation and integration resulted in notable tensions early in the project (Thompson et al. 2017).

Building on this previous scientific work on formative assessments and their focus on ‘beginning at the end’, we apply a Q-methodological approach to make the diverse expectations towards collaboration of the ‘Römerland Carnuntum 2040’ project team visible and tangible. We apply a sound process of integrating theory based with context-specific categories of expectations towards TDR collaboration and transfer them into a comprehensive Q-set (see Sect. 4).

## Case study and project framework

The TDR project ‘Römerland Carnuntum 2040’ is embedded in the region Römerland Carnuntum (in the province of Lower Austria), which is located between the metropolitan areas of Vienna (Austria) and Bratislava (Slovakia) and consists of 30 municipalities and about 80,000 inhabitants. The region benefits from a dynamic demographic and economic development. However, the process of growth and rapid change is accompanied by specific challenges, such as a loss of biodiversity and threats to natural areas, an increase in motorised individual traffic and fine dust pollution, and growing pressure on settlement areas, to name but a few. Traditional planning tools and approaches at the local and regional level, as well as structural policies, fall short in adequately approaching and managing such complex, multidimensional challenges. Therefore, the regional development association ‘Römerland Carnuntum’ (practice partner in the TDR project) aims to experiment and establish new forms of cooperation and self-governance that support a sustainability transition, including new governance formats, collaborative learning, capacity building, and self-empowerment. Different from numerous TDR projects, the practice partner initiated the ‘Römerland Carnuntum 2040’ project and developed a TDR proposal jointly with two universities. The 3-year (2019–2022) TDR project ‘Römerland Carnuntum 2040’ addresses the above-mentioned challenges at several levels: (1) co-creating a joint regional vision for the year 2040 and possible transition pathways; (2) experimenting with deliberative governance formats, by establishing a deliberative forum (Future Council) of regional and local actors and working groups that are closely linked to local and regional decision-makers; and (3) testing new forms of knowledge creation, integration, and learning (e.g. real-word lab, serious games). The TDR project team consists of 13 individuals from six organisations (two universities, regional development association, provincial regional development organisation, one consultant, and a mediator serving as moderator of the meetings) with diverse professional and disciplinary backgrounds. The project also involves meta-research that investigates different aspects of the TDR project and collaboration, and feeds these interim results back into the research process as basis for ongoing reflection and discussion (Christensen et al. 2016; von Wehrden et al. 2019).

## Methods and data: Q-methodology

To bring expectations, assumptions and epistemologies (Hakkarainen et al. 2020) of our TDR collaboration to the surface, we chose to use the Q-methodology, an established explorative, semi-quantitative method for investigating distinctive viewpoints (VPs) of a given population based on inverted factor analysis. Hence, the Q-study reveals the common views of a certain population group on a given topic. It has been used in several contexts such as agriculture and biodiversity (e.g. Braito et al. 2020; Hamadou et al. 2016), resource management (Buckwell et al. 2020; Edgeley et al. 2020; Sardo and Sinnett 2020) or decarbonisation and climate research (Howard et al. 2016).

**Fig. 1:**
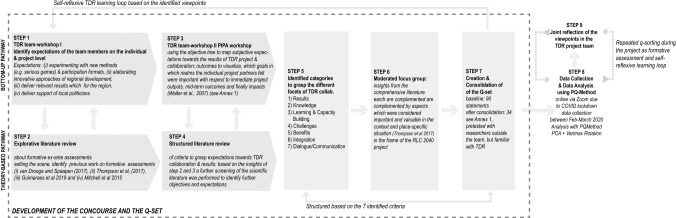
Structure and implementation of data collection and analysis, especially the elaboration of the Q-set (own illustration)

The basic building block of the Q-method is the *Q-set* (or *Q-sample*), a carefully constructed sample of statements (see Table 3), representing a holistic picture of the selected issue (Baker et al. 2017; Watts and Stenner 2005). Since the Q-set forms the basis for data collection, its careful and rigorous construction is crucial for yielding solid results (Baker et al. 2017; Müller and Kals 2004). Figure 1 illustrates the structure and implementation of data collection and analysis: steps 1–7 explain the development of the Q-set; step 8 explains the data collection and analysis; and step 9 the reflection on the results in the TDR project team.

### Q-set development (steps 1–7)

Figure 1 shows in detail the development of the Q-set, which combines a theory-based and a bottom-up approach (Müller and Kals 2004). The deductive, theory-based approach (see Fig. 1) is based on a comprehensive literature review that sets the tone for the investigation. It is complemented by a context-specific bottom-up approach to (1) validate and contextualise the deductive part of the Q-set, and (2) collect and add further aspects and perspectives in team workshops as well as a moderated focus group discussion (see Fig. 1). In an initial team workshop (step 1), an explorative brainstorming approach was used among team members to collect their individual expectations for the TDR project; these are: (1) experimenting with new methods (e.g. serious games) and participation formats, which was frequently mentioned, followed by (2) elaborating innovative approaches for regional development, (3) delivering practice-relevant results, and (4) getting local policy support for the project. Within 4 weeks, another team meeting took place (step 3), where the PIPA objective tree (van Drooge and Spaapen 2017) was applied to structure the subjective expectations for TDR project outcomes and collaboration. The findings are structured along the three PIPA outcome spaces to visualise which goals each project partner considered important in which realms in terms of immediate and mid-term outputs and impacts (Walter et al. 2007).

Both sources of information, the structured literature review and the TDR team workshops (I and II) (see Fig. 1), formed the basis for the creation of the initial Q-set, which is structured along seven literature-based categories (step 6, Alvarez et al. 2010; Guimarães et al. 2019; Mitchell et al. 2015, 2018; Schauppenlehner-Kloyber and Penker 2016; van Drooge and Spaapen 2017). The initial Q-set was validated in a moderated focus group discussion and complemented by topics that had not yet been adequately addressed and were identified by several team members as important topics for the (context- and place-)specific situation (Thompson et al. 2017). The consolidation aimed to (1) merge related statements, and (2) remove redundancies. The final Q-set was pre-tested twice, involving researchers from outside the project team but familiar with TDR. The pretest did not require any modifications other than a minor revision. The statements are originally in German (translated into English for this publication, see Table 3).

### Data collection and analysis (step 8)

The P-sample consists of those individuals, or a selection of them, who are the target group of the survey. In our research all 13 team members of the TDR project ‘Römerland Carnuntum 2040’ (see Table 1) consist of those from two universities (“*Univ. 1*” project lead: four persons, “*Univ. 2*”: three persons), representatives of the regional development association (“*Pract*.”: four persons), and two moderators, who support and facilitate the research collaboration (“*Mod.*”). The data collection took place between February and April 2020; due to the lockdown resulting from the COVID-19 pandemic, face-to-face interviews had to be replaced with online sessions (via Zoom). Therefore, the Q-set and an initial instruction were sent to the interviewees per postal service and the sorting exercise and ex post interview took place via a Zoom meeting. Ethical research standards based on the university’s ethical and legal guidelines, such as free, prior, and informed consent and the implementation of the General Data Protection Regulation (EU) 2016/679 (GDPR 2016), were implemented and performed from the beginning of data collection. The zoom sessions were recorded, transcribed, coded, and analysed (Kuckartz 2016). The Q-sorting exercise consisted of three steps: (1) an introductory question about previous TDR experiences, (2) dividing the 34 statements into three piles (agree, neutral, disagree) as suggested by Stenner et al. (2008), (3) sorting the three piles into the forced normal distribution (on a Likert scale from − 5 “strongly disagree” to + 5 “strongly agree”) (see Table 2), based on the question “To what extent do you agree with the following statement about collaboration within our TDR project?”. The interview partners were asked to share their thoughts and comments while sorting the statements.

**Table 1:** Literature-based and context-specific categories to frame the expectations towards transdisciplinary research collaboration within the project ‘Römerland Carnuntum 2040’ (own illustration)

Category		Explanation	Based on/related to
1	Results	Results of the collaboration refer to initiated and achieved change of the area/everyday world and the improvement of the current situation via the collaboration in the TDR project	PIPA: Alvarez et al. (2010), van Drooge and Spaapen (2017); outcome spaces: Mitchell et al. (2015, 2018); input by TDR team members
2	Knowledge	Stocks and flows of knowledge within the TDR project collaboration and how knowledge moves between the heterogeneous members of the project team	Fam et al. (2020), Mitchell et al. (2015, 2018)
3	Learning and capacity building	Mutual and transformative learning between diverse project team members, capacity building and empowerment beyond the duration of the project	Fam et al. (2020), Mitchell et al. (2018, 2015); input by TDR team members
4	Challenges	Challenges in the collaboration in the TDR project team	Guimarães et al. (2019), Schauppenlehner-Kloyber and Penker (2015, 2016), Thompson et al. (2017)
5	Benefits	Individual (personal or professional) benefits, benefits for the team as well as for the project process and success	Guimarães et al. (2019), Thompson et al. (2017)
6	Integration	Involvement of the various team members, valuing different types of knowledge, ways of knowing and perspectives, organisation of knowledge integration in TDR collaboration	Fam et al. (2020); expertise in research integration: Bammer et al. (2020), Scholz and Steiner (2015)
7	Dialogue and communication	Accessible and multidirectional dialogue (transparency, language, communication) among TDR project team members; leadership communication	Guimarães et al. (2019), Mitchell et al. (2018), Thompson et al. (2017), Wiek (2007)

*PIPA* impact pathways analysis, *TDR* transdisciplinary research

**Table 2:** Viewpoint characteristics (own illustration)

No.	Stakeholder	Viewpoint 1	Viewpoint 2	Self-perceived former TDR experience
1	Univ (sust/td)	**0.7749**	0.2243	Yes
2	Univ (tech/pract)	**0.6850**	0.2420	Yes
3	Univ (sust/td)	**0.7446**	0.0892	Yes
4	Univ (sust/td)	**0.6626**	0.3312	No
5	Univ (sust/td)	**0.7784**	0.3049	Yes
6	Pract1	0.3962	**0.7107**	No
7	Pract2	0.3571	**0.5885**	Yes
8	Univ (tech/pract)	0.1057	**0.4503**	No
9	Univ (tech/pract)	0.0592	**0.4430**	No
10	Pract3	0.0854	**0.4996**	Yes
11	Pract4	0.4216	**0.6957**	Yes
12	Mod1	**0.5054**	**0.6479**	Yes
13	Mod2	**0.4834**	**0.5552**	No
Explained variance (%)		*28*	*23*	

The bold numbers show which team member loads significantly on which viewpoint

To be significant at the *p* < 0.01 level, the factor loading in this study has to be > 0.442. A column was added to show previous experience with TDR based on the self-assessment of TDR members

*Univ* university, *Pract* practitioner, *Mod* moderator, *sust/td* focus on sustainability and TDR and theory, *tech/pract* focus on technical science and practical applicability of research, *TDR* transdisciplinary research

All Q-sorts were photographed and entered into “PQMethod”, a free software for processing Q-studies (Schmolck 2014). In a first step, the calculation of intercorrelations among all Q-sorts reveals similarities and differences between any two Q-sorts (correlations between − 1 and + 1). The closer the value of the correlation coefficient is to 1, the higher the degree of similarity between two Q-sorts (Stenner et al. 2008). In a second step, the Q-sorts are factor-analysed using a Principal Component Analysis (PCA) with a Varimax Rotation, to detect patterns among the Q-sorts and extract distinct *factors* (shared VP) (Schmolck 2014; Stenner et al. 2008; Watts and Stenner 2005). Once factor scores are calculated, each factor is presented in the form of a factor array (see Table 3), which indicates an overall ‘gestalt’ which cannot be further reduced and supports the interpretation of distinction and consensus statements (Stenner et al. 2008).

**Table 3:** Q-statements and factor loadings of the two revealed viewpoints (own illustration)

C	No	Statement	VP1	VP2
1	1	*In my opinion, the most important goal of our TDR project is to improve the actual situation in the region*	− *2*	− *2*
2	2	I am concerned with understanding the theoretical and methodological features of TDR projects	2	0
6	3	*It is easy for me to put my professional expertise behind me and enter into an open dialog within the team*	*0*	*0*
6	4	*I can easily empathise with and understand the priorities and attitudes of other team members*	− *1*	− *1*
2	5	I believe that the most important aspect of our TDR project is learning from each other and reflecting together	4	0
3	6	I think that TDR projects promote more problem awareness and a higher level of ownership of solutions among the participants than more traditional research processes	3	1
7	7	I think that in our TDR project the project leader acts as a coordinator and represents the decisions of the group externally and internally	− 1	− 3
4	8	I experience the high need for coordination resulting from the heterogeneity of the project team as inefficient	− 4	− 3
7	9	I think that despite the collaboration in the project team at eye level, the project management has the control and final decision-making power	− 1	− 2
1	10	The project has failed for me if no direct tangible results (e.g. strategy, plans etc.) are produced	− 3	− 2
6	11	*I think that we work together as equals in our team*	*1*	*1*
2	12	I believe that dealing with complex challenges requires consideration of non-scientific perspectives and knowledge	3	0
7	13	I believe that a common language, that is easy for everyone to understand, is a key success factor	0	2
3	14	I think the strength of transdisciplinary projects is to contribute to social change rather than research	0	− 1
4	15	*I perceive the open-endedness of the project as uncertainty, since it is not clear where the journey will lead*	− ***5***	− ***5***
4	16	*I perceive the project as a risk, because by working with heterogeneous partners, results can occur for which I do not want to take responsibility*	− *4*	− *4*
4	17	I consider it essential that all partners of the TDR project are always involved in every step of the process	− 2	− 1
4	18	*Due to the complexity of a TDR project, a predefined plan with clear goals and structure is essential*	− *2*	− *2*
5	19	*I believe that being involved in our TDR project provides me with recognition and benefits me in my professional environment*	− *3*	− *3*
1	20	It is important to me, that through this project, we develop and establish new ways of doing things in regional development practice	− 1	5
5	21	I believe that the heterogeneity of the participants improves the results of the project (compared to classical research collaborations)	5	4
5	22	The TDR project gives me the opportunity to experience self-efficacy and to actively participate in a transformation process	0	1
7	23	I think, it is important to give the group building process enough time	2	0
5	24	I believe that the result will be worth the extra effort compared to “classically” organised research projects	1	3
4	25	*I believe that for successful project implementation, disclosure and management of conflicts within the team is inevitable*	*2*	*2*
4	26	I believe that all project partners should bear responsibility for the project—from project start to implementation	0	− 1
4	27	Due to the complexity of a TDR project, flexibility in planning, goal formulation and implementation is required	3	4
7	28	I find it quite difficult when my professional expertise is questioned in the group	− 2	− 4
5	29	*I perceive participation in the TDR project as an opportunity for personal development*	*1*	*1*
6	30	I consider the open-endedness of the TDR project an opportunity to experiment and try out new paths	4	3
1	31	It is important to me, to already set the foundation for the transition to the post-project phase during the project	1	2
1	32	The recognition and support of the results by political decision-makers is crucial for our project success	− 3	0
6	33	*I think that openness and tolerance are key qualities for collaboration in transdisciplinary processes*	*2*	*2*
6	34	In our collaboration, I experience the willingness and openness to learn from each other and to get engaged in different work practices	0	3

In italics = similar loadings in VP1 and VP2

*C* category, *TDR* transdisciplinary research, *VP* viewpoint

### Joint reflection on the viewpoints (step 9)

The joint reflection of the results in the TDR project team took place in December 2020. The results were processed, visualised, and presented by the lead author. The reflective discussion was designed as moderated focus groups, and included the following steps: First, both VPs resulting from the Q-method were presented to the team members. This initial presentation was followed by immediate feedback of the group members on whether or to what extent they were surprised by the result. We then split up the TDR team into two subgroups to reflect on (1) what might be the explanations for this result, and (2) how can we learn from these results for our further project collaboration. The reflection was documented via participatory observation and written documentation. After half an hour, the results of both subgroups were presented and discussed with the entire TDR team and documented in a protocol.

## Results

The interview partners found it helpful to pre-sort all 34 statements into three piles (agree, neutral, disagree) and, in a second step, to rank them along the given template. The sorting showed that for all 13 individuals, the number of statements they agreed with exceeded the number they disagreed with (statement of a project team member during the Q-sorting: “*It seems to me, that there are more statements that I agree with than those that I disagree with.*”). The Q-sorting of 34 Q-statements by the 13 project partners resulted in two distinct VPs (significance level 0.442; cumulative explanatory variance of 52%). All Q-sorts were considered factor-defining Q-sorts (Watts and Stenner 2012): each member of the TD project team loaded on one of the two VPs, except for the two moderators, who loaded on both. The results show (Table 2) that the VPs are (1) distributed across the practice and science groups, and (2) not clearly differentiated between the various academic disciplines involved in the TDR team. However, all scientific team members with a disciplinary focus on sustainability and TDR were found to load on VP1, with all practice partners and most scientific team members with a disciplinary focus on technical sciences loading on VP2.

### Viewpoint 1: Transdisciplinary research projects as learning experiments (“learning viewpoint”)

This VP is characterised by a strong focus on learning, collective reflection (statement #5/+ 4), and knowledge exchange and integration among the different team members (#12/+ 3). In this line, representatives of this group considered it important to furnish the process with sufficient time resources for group formation (#23/+ 2). The comparatively high degree of coordination resulting from team diversity is not perceived as inefficient in this VP (#8/− 4).

There is a strong belief that complex real-world challenges require the integration of non-academic knowledge, and other forms of knowing (#12/+ 3), and that the (great) diversity of the group improves project outcomes (#21/+ 5) compared to ‘classical’ disciplinary research approaches. The complexity of real-world challenges and TDR projects is oriented towards openness and open-endedness to results, which is not perceived as uncertainty (#12/− 5). In this sense, TDR collaborations kindle a higher degree of problem awareness and self-responsibility leading to more adequate results compared to “classical” research projects (#6/+ 3). The statement on scientific results, as an important contribution to societal change, was put in the neutral core of the Q-sort (#14/0). At the same time, this VP emphasises the personal interest in experimenting and entering new pathways (#30/+ 4) and focuses on learning theoretical and methodological characteristics/features of transdisciplinarity (#2/+ 2).

Compared to the other one, VP1 does not significantly strive for tangible, specific after-project outcomes such as new strategies, plans, or policies (#10/− 3). Hence, the implementation of new forms, ways, or tools for regional development practice is also not considered pivotal (#20/− 1). Thus, the planning and preparation of the post-project phase (#31/+ 1) is also of less importance than in VP2. The results clearly show that project success is not attributed to the recognition and support of policy makers (#32/− 3).

### Viewpoint 2: Transdisciplinary research as experimental pathways to new practices (“changing practices viewpoint”)

VP2 shows more agreement with the complexity of TDR projects and the associated need for flexibility regarding project planning, target formulation and implementation (#27/+ 4) than VP1. The open-ended project character is considered as an opportunity for experimentation (#30/+ 3). Deviating from VP1, however, a notably higher relevance of practise is noted in VP2: a clear expectation is expressed that the TDR project results in new ways and practices for (sustainable) regional development (#20/+ 5). The additional transaction costs due to coordination efforts compared to “classical” research projects are hence considered worthwhile (#24/+ 3). Therefore, the post-project phase and the carefully planned transition to this phase (#31/+ 2) play an important role in VP2. VP2 also differs from VP1 as it gives slightly more approval to the importance of tangible results (e.g. strategies, policies) (#10/− 2), the support of policy makers for project success (32/0), and the implementation of project results (#20/+ 5).

VP2 also reflects the conviction that there is openness and willingness to learn from each other and to engage with different methods (#34/+ 3). Thus, the project stimulates self-efficacy and the opportunity to actively trigger and shape change processes (#22/+ 1). Consequently, VP2 also emphasises the importance of a common, easily understandable language (#13/+ 2).

Although VP2 values the transdisciplinary project approach and expects it to yield results that improve the current situation and the outcomes, there is only modest interest in deepening conceptual and methodological issues (#2/0), learning processes, and joint reflection (#5/0). Knowledge integration of non-academic partners (#12/0) also scores lower, which might be surprising given that instrumental aspects of TDR (TDR as a means to the end of changed practices) play a more fundamental role in VP2 than in VP1.

### Common and diverging expectations among transdisciplinary research team members

Out of 34 statements, ten have similar loadings in VP1 and VP2 (see Table 3, statements #25, 33, 29, 11, 3, 4, 1, 18, 19, 16, 15, in italics), which means that these aspects of collaboration are rated very similar by all respondents and VPs. Four statements share the highest degree of agreement: (1) “I believe that for successful project implementation, disclosure and management of conflicts within the team is inevitable” (#25/+ 2); (2) “I think that openness and tolerance are key qualities for collaboration in transdisciplinary processes" (#33/+ 2); (3) “I think that we work together as equals in our team” (#11/+ 1); as well as (4) “I perceive participation in the TDR project as an opportunity for personal development” (#29/+ 1). In parallel, there are common disagreements with the statements: (1) “I perceive the open-endedness of the project as uncertainty, since it is not clear where the journey will lead” (#15/− 5); (2) “I perceive the project as a risk, because by working with heterogeneous partners, results can occur for which I do not want to take responsibility” (#16/− 4); and (3) “I believe that being involved in our TDR project provides me with recognition and benefits me in my professional environment” (#19/− 3). Further statements with equivalent loadings on VP1 and VP2 relate to personal attitude to engage in open dialogue with the team (#3/0) as well as empathy and understanding of other team members’ priorities (#4/− 1).

The largest divergence of statements between VP1 and VP2 relates to the development of new practices for regional development (#20, VP1: − 1; VP2: 5), and the role of policy in achieving project success (#32, VP1: − 3, VP2: 0). A third divergent statement refers to the existing collaboration and the perceived willingness and openness to learn from each other and to engage in different working practices within the project team (#34, VP1: 0; VP2: 3). Here, respondents loading on VP1 might see more scope for learning and openness than those representing VP2.

## Discussion

The discussion structures and reflects on the findings of this paper on the two stated research questions, (1) how do the expectations towards TDR collaboration and its outcomes vary across TDR team members?, (2) how can the Q-methodology make varying expectations of TDR collaboration visible and tangible?

### Varying expectations towards collaboration among transdisciplinary research team members

In our project, the Q-methodology resulted in two VPs that emphasise learning on the one hand and experimenting with new regional development practices on the other. In this way, they depict varying expectations towards the TDR collaboration and assumptions, as well as inherent epistemologies (e.g. motivation, goals and varying understanding of project success, validation of findings) (Eigenbrode et al. 2007; Scholz and Steiner 2015). As such, the VPs are close to, but slightly different from, the dichotomy between the realm of science and rigour and the realm of practice and relevance (Pohl et al. 2017). It turned out that TDR project team members share a common understanding of the necessary conditions for TDR collaboration, such as openness and tolerance for collaboration, willingness to address tensions and conflicts, collaboration as equals, and the perception that the open-ended nature of the project is not a threat. Although the TDR project team agrees that diversity within the team leads to better project outcomes (compared to “classical” research approaches), they also share scepticism that TDR collaboration may lead to recognition and benefits in their professional environments. This is in line with Guimarães et al. (2019), who found, that besides the transformative capacity of TDR and its crucial role in addressing future challenges, participation in TDR is not yet recognised as an essential skill and capacity for a practical or scientific career; for the latter, it is even seen as a possible obstacle (Fam et al. 2020; Guimarães et al. 2019). Apart from these commonalities, there are some major discrepancies in the expected outcomes of TDR collaboration, i.e. on the one hand, the adoption of new practices for regional development and, on the other hand, the importance given to the support of policymakers.

It is primarily the practice partners who load on only VP2 (‘changing practices’), while scientific team members loaded on both VPs (see Table 2). Contrary to the literature (Guimarães et al. 2019; Lang et al. 2012; Mitchell et al. 2015; Thompson et al. 2017), previous TDR experience (see Table 2) does not help explain the allocation of team members to one VP or the other in our Q-study: 5 of the 13 TDR project partners from science and practice had no prior TDR project experience. At the same time, they load on both VPs, which is also true for the team members with TDR experience. Prior TDR experience also does not explain the moderators loading on both VPs, as one has prior experience, while the second does not. Although prior TDR experience does not help explain differences in expectations in our TDR project, we can generalise from our single case that future research across multiple TDR projects is needed to provide a clearer picture on the link between prior TDR experience and expectations.

Disciplinary background is another aspect that is highlighted in literature as a possible explanation for divergent expectations in TDR. Hirsch Hadorn et al. (2008) and Guimarães et al. (2019) state that disciplinary backgrounds carry the potential to create path dependencies by shaping the scientific approach and forming the initial institutional and cognitive units in academia. The scientists of the TDR project team have a social sciences, spatial research, regional development, or spatial planning background, which shows some sensitivity to real-world problems compared to other disciplines (e.g. natural sciences, see Thompson et al. 2017), whose prevailing epistemological perspectives are more challenging for TDR collaboration. Nevertheless, we identified two distinct groups of scientists within our TDR project (see Table 2): one is more concerned with sustainability and transdisciplinary research, and thus with academic publications, and a second with a focus on technical sciences aiming at the applicability and relevance for practice. Along theses disciplinary lines, scientists engaged in sustainability and transdisciplinary research loaded on VP1, while scientists focused on the practical applicability of their research as well as practice partners loaded on VP2 (with one exception) (see Table 1). This observation in our TDR project confirms that the selection of disciplines might be critical for a balanced representation and implementation of scientific progress while developing “workable” practical solutions (Lang et al. 2012; Thompson et al. 2017). Furthermore, we link to the considerations of Wittmayer and Schäpke (2014), who propose five core roles for action research in the broader context of sustainability: change agent, knowledge broker, reflective scientist, self-reflexive scientist, and process facilitator. We add that these roles should be applied not only to the scientists involved in TDR but also to the entire project team, including practice partners. Since the two facilitators in our sample load on both VPs, the reflections of the findings might further contribute to this debate. According to the team reflections, facilitators or ‘epistemediators’ who are able to bridge different VPs can reach their full potential (Hirsch Hadorn et al. 2008).

Although at first glance, the two VPs seemed pleasing in the team reflection as TDR collaborations are considered effective when they bring relevant results for both sides (Binder et al. 2015; Gibbons and Nowotny 2001; Gibbons et al. 1994; Lang et al. 2012; van Drooge and Spaapen 2017), the results presented also harbour tensions. First, we detected an “I – We” tension (Fam et al. 2020), which occurs between the high priority given to the “collaboration as equals and openness and tolerance towards every opinion” on the one hand, and the low personal willingness/capability to take back one’s own disciplinary view or to engage with other opinions on the other hand. Two further tensions refer to “disciplinary versus TDR” and “research versus learning”: while one subgroup attributes project success to the establishment of new ways/forms of regional development and the commitment of local and federal policymakers to support the project, the other subgroup considers the degree of learning as a measure for a successful project. Another tension is the perceived reluctance in the TDR collaboration towards mutual learning, engaging in different work practices, and thus appreciating different forms of knowing. This tension between “disciplinary versus TDR” might indicate a modest ‘shared understanding’ of the different research cultures and backgrounds involved beyond one’s own (see also Barth et al. 2020; Scholz and Steiner 2015), which jeopardises the abandonment of disciplinary path dependencies and impedes further knowledge integration. Based on the revealed tensions, another one was discussed in the team reflection, namely the question of resources. As Mitchell et al. (2015) also stated, the prioritisation of objects within TDR collaboration goes hand in hand with the allocation of resources, particularly time and project funds. In this way, the elaborated VPs provided individuals the opportunity to learn about their differences and enable TDR ‘collaboration without consensus’ (Carlile 2004).

The tensions elucidated in the Q-analysis necessitate the role of the ‘epistemediator’, who mediates between different epistemologies and facilitates the process of joint knowledge generation and integration (Wiek 2007). Furthermore, fostering communication between alternative knowledge communities as well as deliberate social unlearning, especially by academics, are consequences of managing those tensions (Alonso-Yanez et al. 2019). The different VPs identified in the TDR project ‘Römerland Carnuntum 2040’ raised awareness of the diverse expectations towards the TDR collaboration and highlighted the need for continuous monitoring and evaluation (Alonso-Yanez et al. 2019; Pohl et al. 2017; Schön 1983; Thompson et al. 2017). In the project, the accompanying research fulfils the task of continuous monitoring, data collection, and analysis as a basis for further team reflection, and the two moderators, who load on both VPs, can support the balancing of expectations, resources, and priorities in the team meetings.

### The Q-method as formative assessment to elevate varying expectations towards transdisciplinary research

The two VPs helped us identify disciplinary and individual subliminal expectations for further team reflection, confirming that formative assessments are useful in early project phases.

We agree with Stenner et al. (2008) that the Q-methodology has proven useful in producing meaningful results for a small population (in our case 13 members of the project team). The deductive–inductive process of identifying and consolidating Q-statements considered a wide variety of aspects of TDR collaboration and its outcomes. The resulting list of statements may be a valuable basis for in situ formative assessment of other TDR project teams. They could be used not only in a Q-method approach, but also in a larger N-survey. TDR projects require context sensitivity and place specificity (Thompson et al. 2017). Thus, Q-statements should be contextualised through a project- and place-specific lens. The proposed dual approach, combining a theory-based (deductive) and a practise-based (inductive) strand for assembling the Q-sort, has proven useful. VP “learning” and VP “changing practices” are also to be interpreted from this perspective. Precisely because the Q-statements and the resulting VPs are context- and place-specific and all team members could equally contribute their subjective expectations, the project team valued the results as a tangible and concrete basis for team reflection. One disadvantage of the Q-method is the amount of time and effort that has to be invested in developing the Q-statements. This is justified if this semi-quantitative method is also used for further interim and ex post assessments as it has the potential for continuous evaluation (Lang et al. 2012), and in doing so supports team reflection and TDR leadership (Scholz and Steiner 2015).

From our research, we can deduce some promising lines for further research, namely (1) the potential of the Q-method as an evaluation tool to track change and social learning within the TDR team throughout the TDR process, and (2) the need of going beyond single case studies to multiple case studies, or larger N-studies using the Q-method or other methods to understand the overall breadth of VPs in TDR, and the underlying explanatory variables such as academic/non-academic background, disciplinary affiliation, previous TDR experience, etc.

## Conclusions

The contributions of this paper are twofold: on the one hand, we provide a validated set of Q-statements for a formative assessment to externalise TDR project team members’ implicit expectations, assumptions, and epistemologies regarding their expectations towards TDR collaboration and outcomes. On the other hand, we present the Q-methodology as a viable approach to uncover divergent VPs as visible, tangible, and enunciable differences, that need to be acknowledged in team meetings when allocating resources and planning next project steps.
